# Pre-transplant HLA Antibodies and Delayed Graft Function in the Current Era of Kidney Transplantation

**DOI:** 10.3389/fimmu.2020.01886

**Published:** 2020-08-26

**Authors:** Christian Morath, Bernd Döhler, Florian Kälble, Luiza Pego da Silva, Fabian Echterdiek, Vedat Schwenger, Stela Živčić-Ćosić, Nataša Katalinić, Dirk Kuypers, Peter Benöhr, Marion Haubitz, Malte Ziemann, Martin Nitschke, Florian Emmerich, Przemyslaw Pisarski, Hristos Karakizlis, Rolf Weimer, Andrea Ruhenstroth, Sabine Scherer, Thuong Hien Tran, Arianeb Mehrabi, Martin Zeier, Caner Süsal

**Affiliations:** ^1^Division of Nephrology, Heidelberg University Hospital, Heidelberg, Germany; ^2^Institute of Immunology, Heidelberg University Hospital, Heidelberg, Germany; ^3^Department of Nephrology and Autoimmune Diseases, Transplantation Center, Klinikum Stuttgart, Stuttgart, Germany; ^4^Department of Nephrology, Dialysis and Kidney Transplantation, Department of Internal Medicine, Clinical Hospital Center Rijeka, Faculty of Medicine, University of Rijeka, Rijeka, Croatia; ^5^Tissue Typing Laboratory, Clinical Institute of Transfusion Medicine, Clinical Hospital Center Rijeka, Faculty of Medicine, University of Rijeka, Rijeka, Croatia; ^6^Department of Nephrology and Renal Transplantation, University Hospitals Leuven, Leuven, Belgium; ^7^Department of Nephrology and Hypertension, Center for Internal Medicine and Medical Clinic III, Klinikum Fulda, Fulda, Germany; ^8^Institute of Transfusion Medicine, University Hospital of Schleswig-Holstein, Lübeck, Germany; ^9^Medical Clinic 1, Transplantation Center, University of Lübeck, Lübeck, Germany; ^10^Institute for Transfusion Medicine and Gene Therapy, University Medical Center, University of Freiburg, Freiburg, Germany; ^11^Department of General and Digestive Surgery, University Medical Centre Freiburg, Freiburg, Germany; ^12^Department of Internal Medicine, University of Giessen, Giessen, Germany; ^13^Department of General and Transplant Surgery, University Hospital Heidelberg, Heidelberg, Germany

**Keywords:** renal transplantation, HLA antibodies, donor-specific antibodies, delayed graft function, biopsy-proven rejections, antibody-mediated rejections

## Abstract

Delayed graft function (DGF) occurs in a significant proportion of deceased donor kidney transplant recipients and was associated with graft injury and inferior clinical outcome. The aim of the present multi-center study was to identify the immunological and non-immunological predictors of DGF and to determine its influence on outcome in the presence and absence of human leukocyte antigen (HLA) antibodies. 1,724 patients who received a deceased donor kidney transplant during 2008–2017 and on whom a pre-transplant serum sample was available were studied. Graft survival during the first 3 post-transplant years was analyzed by multivariable Cox regression. Pre-transplant predictors of DGF and influence of DGF and pre-transplant HLA antibodies on biopsy-proven rejections in the first 3 post-transplant months were determined by multivariable logistic regression. Donor age ≥50 years, simultaneous pre-transplant presence of HLA class I and II antibodies, diabetes mellitus as cause of end-stage renal disease, cold ischemia time ≥18 h, and time on dialysis >5 years were associated with increased risk of DGF, while the risk was reduced if gender of donor or recipient was female or the reason for death of donor was trauma. DGF alone doubled the risk for graft loss, more due to impaired death-censored graft than patient survival. In DGF patients, the risk of death-censored graft loss increased further if HLA antibodies (hazard ratio HR=4.75, *P* < 0.001) or donor-specific HLA antibodies (DSA, HR=7.39, *P* < 0.001) were present pre-transplant. In the presence of HLA antibodies or DSA, the incidence of biopsy-proven rejections, including antibody-mediated rejections, increased significantly in patients with as well as without DGF. Recipients without DGF and without biopsy-proven rejections during the first 3 months had the highest fraction of patients with good kidney function at year 1, whereas patients with both DGF and rejection showed the lowest rate of good kidney function, especially when organs from ≥65-year-old donors were used. In this new era of transplantation, besides non-immunological factors, also the pre-transplant presence of HLA class I and II antibodies increase the risk of DGF. Measures to prevent the strong negative impact of DGF on outcome are necessary, especially during organ allocation for presensitized patients.

## Introduction

Acute renal injury early after transplantation can lead to delayed graft function (DGF), increase the immunogenicity of the tissue and result in immunological rejection episodes requiring treatment (1).

The reported incidence of DGF after deceased donor kidney transplantation varies between 5 and 50% and continues to grow as kidneys from elderly donors are increasingly used due to organ shortage (2–5). DGF was reported to have a negative impact on 12-month graft function (6) and longterm graft survival, almost doubling the risk of 5-year graft loss according to a recent study (7). Interventions to reduce the incidence of DGF, such as donor dopamine infusion or machine perfusion during organ removal and transport, are still experimental and there is no approved therapy to reduce or treat DGF (8). Therefore, there is a great interest in the early detection of procurement-, donor- and recipient-related risk factors of DGF to ensure optimal treatment for patients at risk. In addition to non-immunological factors, such as donor brain death, prolonged cold ischemia time and donor and recipient age, involvement of immunological factors has also been reported in the development of DGF (9, 10). Earlier data from the Serum Study of the Collaborative Transplant Study (CTS) indicated that adverse events in deceased donor kidney transplantation, such as no immediate function and rejection episodes during the first 3 months post-transplant, are associated with pre-transplant presence of alloantibodies against human leukocyte antigens (HLA) (11). Patients with these early adverse events showed significantly impaired graft survival rates. In the meantime, small single-center studies indicated that donor-specific HLA antibodies (DSA) and rejection episodes are particularly detrimental in patients with DGF, while more recent large-scale studies on an involvement of DSA in DGF are lacking (12, 13).

Sensitive antibody detection techniques have become routine since 2008 and this might have diminished the involvement of overlooked HLA antibodies in DGF. On the other hand, the risk of DGF is expected to have increased due to the growing use of kidneys from elderly donors. The aim of the current study was to identify the immunological and non-immunological predictors of DGF and to determine the alloantibody-dependent influence of DGF on post-transplant outcomes in a large cohort of patients transplanted at 8 different transplant centers in the recent 2008–2017 period.

## Materials and Methods

### Study Population

The eight participating centers provided a pre-transplant serum on patients enrolled in the prospectively designed CTS Serum Study (www.ctstransplant.org) and completed a questionnaire 3 months post-transplant which contained the following queries: immediate function within the first 24 h after transplantation (e.g., >500 ml transplant urine), dialysis during the first post-transplant week (except for single dialysis for hyperkalemia), biopsy-proven rejection during the first 3 months, including the time and type of first rejection (borderline, T-cell-mediated, antibody-mediated or mixed T-cell- and antibody-mediated). The work of the CTS is approved by the Ethics Committee of the Medical Faculty of Heidelberg University (No. 083/2005) and performed in accordance with the World Medical Association Declaration of Helsinki Ethical Principles in the currently valid version (14).

The HLA antibody screening was performed centrally in Heidelberg, using the AbScreen I and II ELISA kits of Biotest (Dreieich, Germany) which detected HLA class I and class II antibodies of the IgG isotype. Based on previous findings, an optical density (OD) of more than or equal to 300 was used as cut-off for anti-HLA positivity (15). As this kit was discontinued by the manufacturer, the LABScreen™ Mixed kit of Thermofisher/OneLambda (West Hills, CA, US) was used in 30% (513/1724) of the sera for detection of IgG HLA antibodies, following adjustment of the positivity cut-off to the normalized background ratio of ≥20 which resembles the positivity level of AbScreen ELISA.

DGF, delayed graft function; ESRD, end-stage renal disease; PRA, panel-reactive antibodies; DSA, donor-specific HLA antibodies. Bold means statistically significant.

DGF was defined as either no graft function during the first 24 h and/or dialysis during the first week (except for single dialysis for hyperkalemia) after transplantation (16). Adult patients (≥18 years) on whom we obtained a pre-transplant serum and a complete 3-months questionnaire and who received a kidney-only transplant from a deceased donor between January 1, 2008 and December 31, 2017 and had a functioning graft ≥8 days post-transplant were analyzed. The information obtained from the questionnaires was entered into the CTS database and connected with additional information on the transplants. In 757 cases (44%), we obtained from the participating centers information on the presence or absence of pre-transplant DSA as determined by single antigen bead technique.

### Statistical Analysis

All cause graft, death-censored graft, and patient survival were analyzed from day 8 to the end of year 3 after transplantation. Multivariable Cox regression analysis was performed to account for the possible influence of the following confounders on graft survival: transplant year, transplant number, recipient age, recipient and donor sex and combination, diabetes mellitus as cause of end-stage renal disease, donor age, cold ischemia time, time on dialysis, HLA A+B+DR mismatches, general evaluation of the patient by the physician, latest panel-reactive antibody, donor history of hypertension, trauma as cause of donor death, donation after cardiac death, other causes of marginal donor, e.g., increased serum creatinine, antibody induction therapy, pre-transplant HLA class I and II antibodies and their combination, pre-transplant DSA, and DGF. Survival rates were illustrated using the Kaplan-Meier method.

Significant predictors of DGF and the influence of DGF together with HLA antibodies or DSA on biopsy-proven rejections during days 8–90 post-transplant were determined by multivariable logistic regression analysis, using the same confounders as in the Cox regression analysis. A stepwise backwards elimination of non-significant confounders was applied in the multivariable regression analysis. The software package IBM SPSS Statistics 25 (SPSS Inc, Chicago, IL, US) was used.

## Results

### Predictors of DGF

**Figure 1:**
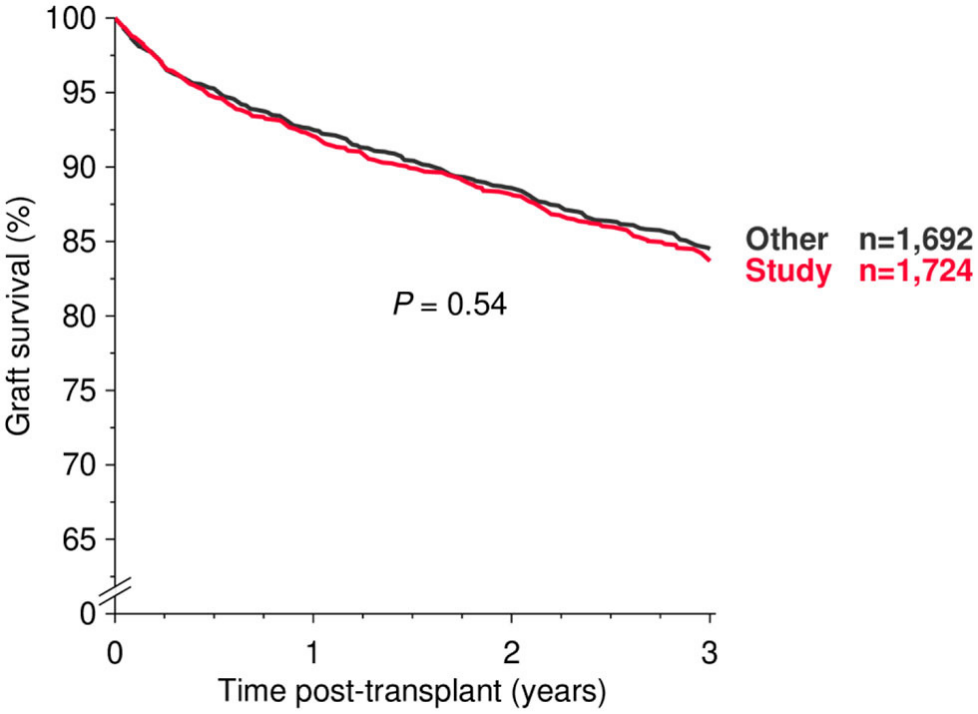
All-cause graft survival during the first 3 post-transplant years in study patients and all other patients who received a deceased-donor kidney transplant at the participating centers during 2008–2017 (log rank *P* value is shown).

A total of 1,724 patients from 8 centers who received a deceased donor kidney transplant between 2008 and 2017 and on whom a pre-transplant serum sample and a 3-months questionnaire on early adverse events was obtained in the framework of the prospective Serum Study of CTS (www.ctstransplant.org) was analyzed. These patients represented a random sample and a graft survival rate which was identical with that observed in 1,692 patients who were not included in the study, but received a deceased donor kidney transplant over the same time period at the same centers (Figure 1).

Table 1 shows the demographics of the study cohort. The patients were stratified according to whether they had DGF (*n* = 482, 28.0%) or not (*n* = 1,242, 72.0%). DGF was more frequent in the more recent 2013–2017 than in the earlier 2008–2012 period (240/748, 32.1 vs. 242/976, 24.8%, *P* < 0.001). The mean of donor age was higher (56.9 vs. 52.4 years, *P* < 0.001) and the mean of cold ischemia time was longer (14.7 vs. 14.0 h, *P* = 0.042) in patients with DGF than in patients without DGF. Patients who developed DGF were more likely to be male (67.2 vs. 60.4%, *P* = 0.009) and older (56.1 vs. 54.2 years, *P* = 0.004). Furthermore, they had a longer dialysis time (6.8 vs. 6.0 years, *P* < 0.001) and more frequently a poor HLA match (5–6 HLA-A+B+DR mismatches: 16.6% vs. 12.8%, *P* = 0.019) and diabetes mellitus as cause of ESRD (10.6 vs. 6.8%, *P* = 0.010).

**Table 1:** Demographics of study patients, *n* (%) or mean ± SD.

**Characteristic**	**Unknown**	**No DGF**	**With DGF**	***P* value**
	**(%)**	***n* = 1,242**	***n* = 482**	
**TRANSPLANT YEAR**	–			**<0.001**
2008–2012		734 (59)	242 (50)	
2013–2017		508 (41)	240 (50)	
**TRANSPLANT NUMBER**	–			0.17
First transplant		1,059 (85)	398 (83)	
Re-transplant		183 (15)	84 (17)	
**RECIPIENT GENDER**	–			**0.009**
Female		492 (40)	158 (33)	
Male		750 (60)	324 (67)	
**RECIPIENT AGE (YEARS)**	–	54.2 ± 13.0	56.1 ± 12.5	**0.004**
**DONOR GENDER**	–			0.11
Female		618 (50)	219 (45)	
Male		624 (50)	263 (55)	
**DONOR AGE (YEARS)**	–	52.4 ± 16.0	56.9 ± 14.3	**<0.001**
**COLD ISCHEMIA TIME (HOURS)**	–	14.0 ± 4.7	14.7 ± 5.7	**0.042**
**TIME ON DIALYSIS (YEARS)**	–	6.0 ± 4.1	6.8 ± 4.6	**<0.001**
**DIABETES MELLITUS AS CAUSE OF ESRD**		85 (7)	51 (11)	**0.010**
**HLA-A+B+DR MISMATCHES**	–			**0.019**
0–1		243 (20)	78 (16)	
2–4		840 (68)	324 (67)	
5–6		159 (13)	80 (17)	
**CYTOTOXIC PRA**	–			0.66
≤5%		1,132 (91)	436 (90)	
>5%		110 (9)	46 (10)	
PRE-TRANSPLANT HLA ANTIBODIES^*^	–			0.066
I neg, II neg		1,034 (83)	393 (82)	
I neg, II pos		61 (5)	21 (4)	
I pos, II neg		76 (6)	24 (5)	
I pos, II pos		71 (6)	44 (9)	
**PRE-TRANSPLANT DSA**	56			0.27
No		481 (85)	157 (82)	
Yes		84 (15)	35 (18)	

* ELISA or LABScreen Mixed.

In the multivariable logistic regression analysis, donor age ≥70 years and simultaneous presence of HLA class I and II antibodies before transplantation were the strongest predictors of DGF (odds ratio [OR]=2.32 and 1.93, *P* < 0.001 and 0.002, respectively; Table 2). They were followed by donor age 60–69 years (OR = 1.64, *P* = 0.001), diabetes mellitus as cause of end-stage renal disease (OR = 1.62, *P* = 0.012), cold ischemia time ≥18 h (OR = 1.60, *P* < 0.001), pre-transplant time on dialysis >5 years (OR = 1.48, *P* < 0.001) and donor age 50–59 years (OR = 1.46, *P* = 0.009). A reduced risk of DGF was found when the cause of donor death was trauma (OR = 0.61, *P* = 0.002) or when recipient or donor gender was female (OR = 0.73, *P* = 0.008, and OR = 0.74, *P* = 0.007, respectively).

**Table 2:** Significant predictors of delayed graft function as result of multivariable logistic regression.

**Predictor**	**OR**	**95 % CI**	***P***
Female recipient	0.73	0.58–0.92	**0.008**
Female donor	0.74	0.59–0.92	**0.007**
Donor 50–59 years	1.46	1.10–1.95	**0.009**
Donor 60–69 years	1.64	1.22–2.22	**0.001**
Donor ≥70 years	2.32	1.65–3.26	**<0.001**
Trauma as cause of donor death	0.61	0.45–0.83	**0.002**
Cold ischemia time ≥18 h	1.60	1.23–2.09	**<0.001**
Diabetes mellitus as cause of ESRD	1.62	1.11–2.37	**0.012**
Time on dialysis >5 years	1.48	1.18–1.86	**<0.001**
HLA class I and II AB pos	1.93	1.28–2.92	**0.002**

Odds ratios (OR) with 95%-confidence intervals (CI) are shown.

ESRD, end-stage renal disease; AB pos, antibody-positive. Bold means statistically significant.

### Influence of DGF and Pre-transplant HLA Antibodies on 3-Year Graft and Patient Survival

**Figure 2:**
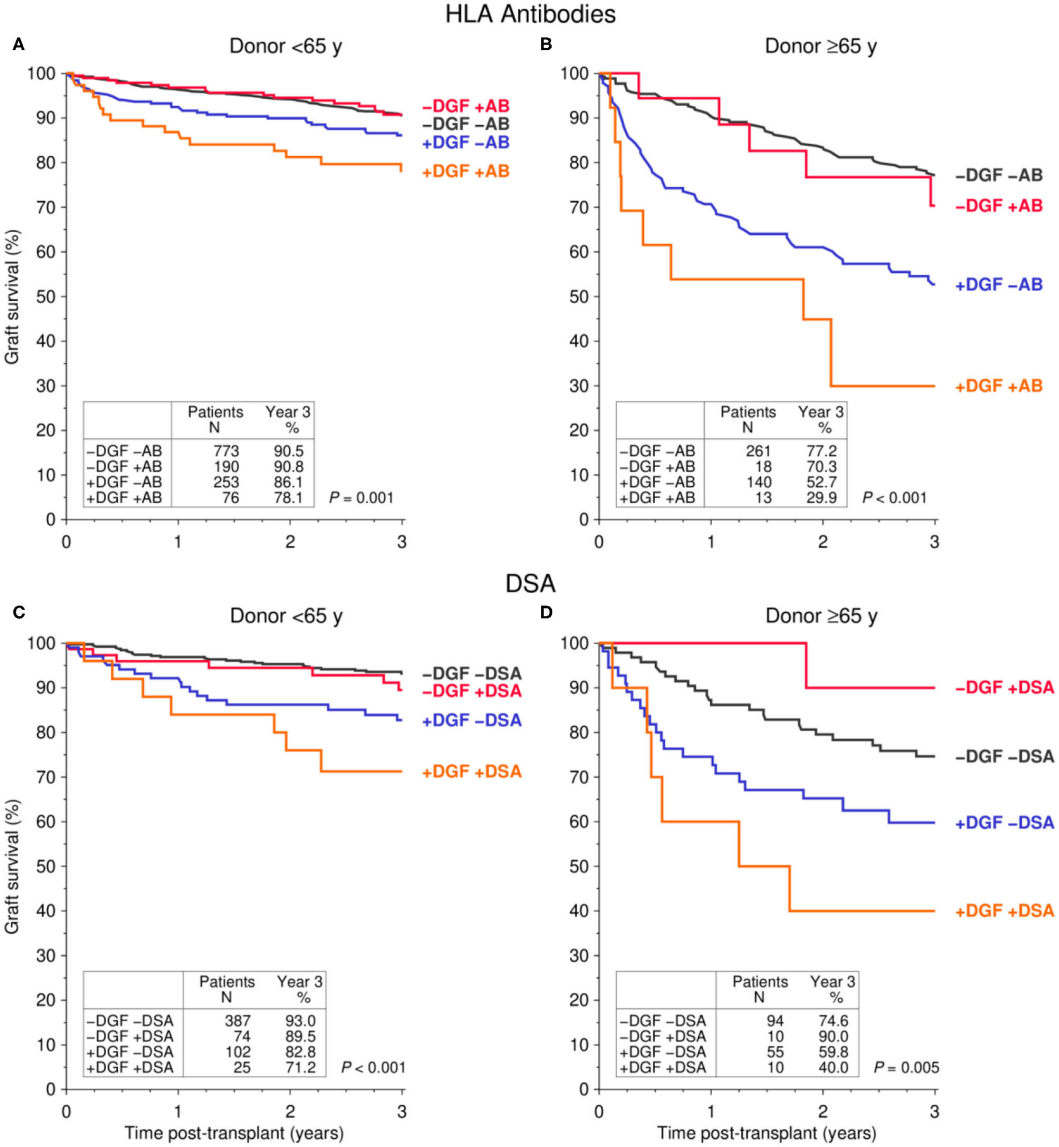
Influence of pre-transplant **(A,B)** HLA antibodies (AB) and **(C,D)** donor-specific HLA antibodies (DSA) in combination with delayed graft function (DGF) on graft survival during the first 3 post-transplant years stratified by donor age [**(A,C)** <65y, **(B,D)** ≤65y] (log rank *P* values are shown).

Figure 2 shows the influence of DGF on 3-year graft survival in patients with and without pretransplant HLA antibodies (Figures 2A,B) or donor-specific HLA antibodies (DSA, Figures 2C,D).

DGF was observed more frequently in patients who received a kidney transplant from ≥65- than <65 year-old-donors (153/432, 35.4% vs. 329/1,292, 25.5%; *P* < 0.001). Only 8.5% (13/153) of DGF patients who received a transplant from a ≥65-year-old donor had HLA antibodies prior to transplantation, as compared to the much higher 23.1% rate (76/329, *P* < 0.001) in patients with transplants from a <65-year-old donor. Because of this ambiguous distribution of variables in the different donor age groups, we stratified the univariate results according to donor age (Figures 2A,C: <65-year-old donor, Figures 2B,D: ≥65-year-old donor). Of note, due to a strong correlation of donor and recipient age, presumably as a result of age matching, e.g., in the Eurotransplant Senior Program, recipients of organs from <65-year-old donors were with a median of 52 years (interquartile range [IQR] 43–59 years) significantly younger than recipients of a graft from a ≥65 year-old donor (median 68 years, IQR 65–70 years).

For both donor age groups (<65- and ≥65-year-old), overall graft survival in patients without DGF was equally good, regardless of whether or not these patients had HLA antibodies (Figures 2A,B) or even DSA (Figures 2C,D) prior to transplantation. In contrast, the 3-year graft survival was significantly reduced in patients with DGF, even in the absence of pre-transplant HLA antibodies or DSA. The worst graft survival was observed in patients who had HLA antibodies or DSA before transplantation and developed DGF.

These results were confirmed in multivariable Cox regression analyses (Table 3). The overall graft survival was significantly reduced in patients with DGF, more due to impaired death-censored graft than patient survival. Compared to DGF-negative patients, the risk for death-censored graft loss was 2.37-fold higher in DGF-positive patients in the absence and 4.75-fold higher in the presence of pretransplant HLA antibodies (*P* < 0.001 for both). An even stronger increase of risk from 2.97- to 7.39-fold was observed in DGF-positive patients with pre-transplant DSA (*P* < 0.001 for both).

**Table 3:** Results of multivariable Cox regression for the influence of delayed graft function (DGF), HLA antibodies (AB), and donor-specific antibodies (DSA) on survival during first 3 post-transplant years.

**Confounder**	**N**	**HR**	**95 % CI**	***P***
**ALL CAUSE GRAFT SURVIVAL**				
**HLA Antibodies**				
–DGF –AB	1,034	ref.		
–DGF +AB +DGF –AB +DGF +AB	208 393 89	1.13 2.02 3.44	0.71–1.80 1.55–2.65 2.20–5.36	0.62 ** <0.001** ** <0.001**
**DSA**				
–DGF –DSA	481	ref.		
–DGF +DSA +DGF –DSA +DGF +DSA	84 157 35	1.04 2.16 3.94	0.49–2.21 1.40–3.33 2.13–7.30	0.92 ** <0.001** ** <0.001**
**DEATH-CENSORED GRAFT SURVIVAL**				
**HLA Antibodies**				
–DGF –AB	1,034	ref.		
–DGF +AB +DGF –AB +DGF +AB	208 393 89	1.43 2.37 4.75	0.80–2.58 1.64–3.42 2.74–8.22	0.23 ** <0.001** ** <0.001**
**DSA**				
–DGF –DSA	481	ref.		
–DGF +DSA +DGF –DSA +DGF +DSA	84 157 35	1.32 2.97 7.39	0.49–3.57 1.59–5.55 3.50–15.6	0.59 ** <0.001** ** <0.001**
**PATIENT SURVIVAL**				
**HLA Antibodies**				
–DGF –AB	1,034	ref.		
–DGF +AB +DGF –AB +DGF +AB	208 393 89	1.05 1.78 1.75	0.54–2.06 1.24–2.57 0.79–3.84	0.88 **0.002** 0.17
**DSA**				
–DGF –DSA	481	ref.		
–DGF +DSA +DGF –DSA +DGF +DSA	84 157 35	0.95 1.66 1.35	0.33–2.74 0.92–2.98 0.40–4.51	0.92 0.093 0.63

*Hazard ratios (HR) with 95%-confidence intervals (CI) are shown. Bold means statistically significant*.

### Influence of DGF and Pre-transplant HLA Antibodies on Biopsy-Proven Rejection Episodes During the First 3 Post-transplant Months

**Figure 3:**
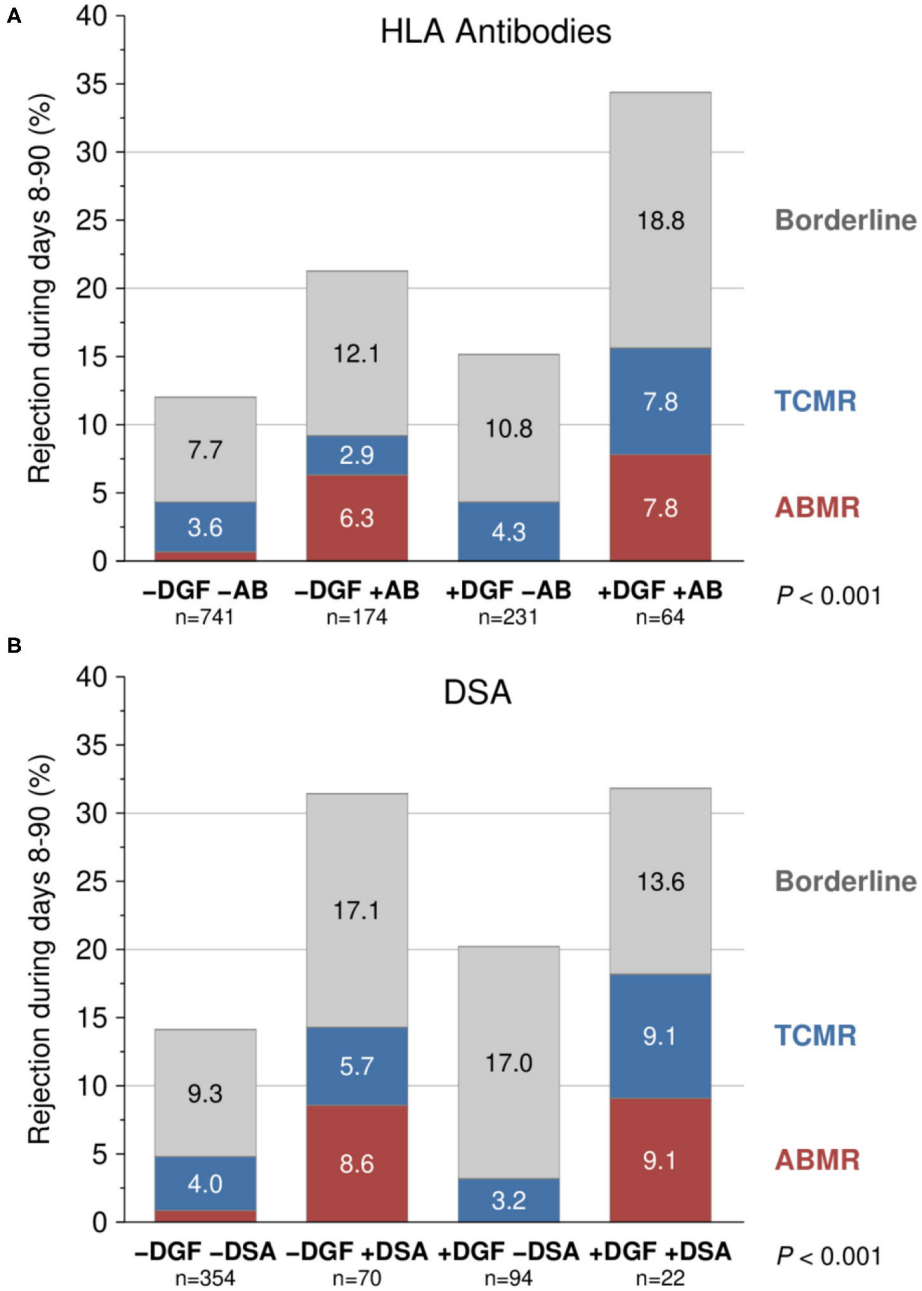
Influence of pre-transplant **(A)** HLA antibodies (AB) and **(B)** donor-specific HLA antibodies (DSA) in combination with delayed graft function (DGF) on biopsy-proven rejection episodes during days 8–90 post-transplant (*P* value of chi-squared test is shown). Transplantations from <65-year-old donors were analyzed. Only the pairwise differences regarding DGF are not significant (1st column vs. 3rd column *P* = 0.26 and 0.15, 2nd column vs. 4th column *P* = 0.14 and 0.94; 1st vs. 2nd column *P* < 0.001; 3rd vs. 4th column *P* < 0.001 and 0.015). TCMR, T-cell-mediated rejection; ABMR, antibody-mediated rejection.

Figure 3 illustrates the incidence of biopsy-proven rejection episodes from day 8 to 90 post-transplant for patients who received a kidney from a <65-year-old donor. Due to low patient numbers, generation of robust results for donors aged ≥65 years was not possible. Irrespective of whether the patients developed DGF or not, significantly higher rates of rejections, especially antibody-mediated rejections, were seen in patients with pre-transplant HLA antibodies or DSA than in patients without such antibodies. The multivariable analysis confirmed the univariate results with higher ORs for development of rejection in patients with pre-transplant HLA antibodies or DSA (Table 4). This association was statistically significant for all HLA antibody-positive groups and there was also a trend toward significance for DSA-positive patients with DGF (OR = 2.18, *P* = 0.053). DGF alone had no significant effect on the occurrence of rejections from day 8 to 90 after transplantation. To avoid a statistical bias, rejections during the first 7 days were not considered, most probably resulting in an underestimation of the proportion of rejections in patients with DGF. Indeed, 35% and 30% of rejections in DGF-patients with or without HLA antibodies, respectively, were observed during the first 7 days post-transplant, as compared to the much lower 25 and 18% rates in DGF-negative patients with and without HLA antibodies, respectively.

**Table 4:** Results of logistic regression for the influence of HLA antibodies (AB) and donor-specific HLA antibodies (DSA) in combination with delayed graft function (DGF) on biopsy-proven rejections during days 8–90 post-transplant.

**Predictor**	**N**	**OR**	**95 % CI**	***P***
**HLA ANTIBODIES**				
–DGF –AB	1,034	ref.		
–DGF +AB +DGF –AB +DGF +AB	208 393 89	1.76 1.29 2.41	1.20–2.59 0.93–1.77 1.45–4.01	**0.004** 0.12 **<0.001**
**DSA**				
–DGF –DSA	481	ref.		
–DGF +DSA +DGF –DSA +DGF +DSA	84 157 35	2.56 1.53 2.18	1.51–4.36 0.97–2.43 0.99–4.80	**<0.001** 0.068 0.053

Odds ratios (OR) with 95%-confidence intervals (CI) are shown. Bold means statistically significant.

### One-Year Kidney Graft Function Depending on DGF and Biopsy-Proven Rejection Episodes During the First 3 Post-transplant Months

**Figure 4:**
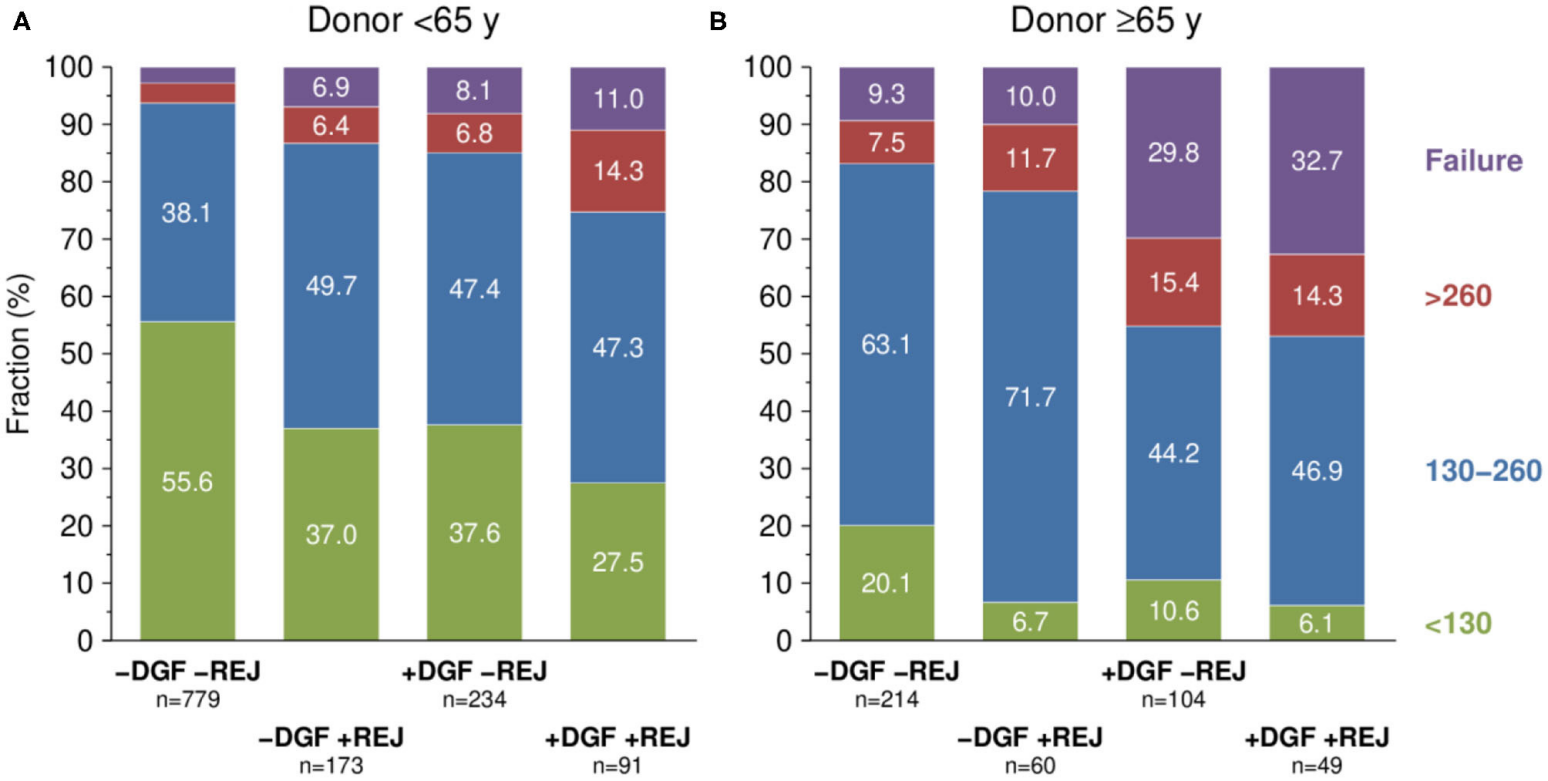
Serum creatinine at 1 year post-transplant (μmol/L) depending on delayed graft function (DGF) and biopsy-proven rejection during first 3 months (REJ) stratified by donor age [**(A)** <65y, **(B)** ≤65y]. *P* values of chi-squared test with trend <0.001. Y, years of age.

The impact of DGF and rejections on serum creatinine at year 1 post-transplant, as stratified by donor age, is shown in Figure 4. Recipients of kidney allografts from <65-year-old deceased donors without DGF and without rejections during days 8–90 post-transplant had with 55.6% the highest fraction of patients with good kidney function at year 1 (creatinine <130 μmol/L) followed by patients with only DGF (37.6%) and only rejections (37.0%). Among patients with both DGF and rejections (REJ), the percentage of patients with good kidney function was an extremely low 27.5%; accompanied by a high graft failure rate of 11.0% during the first post-transplant year (Figure 4A).

When recipients of kidneys from ≥65-year-old deceased donors were analyzed, the fraction of patients with good kidney function at year 1 post-transplant was, overall, strikingly low with 20.1% in –DGF/–REJ, 10.6% in +DGF/–REJ, 6.7% in –DGF/+REJ, and 6.1% in +DGF/+REJ cases. Conversely, the rate of graft failure during the first post-transplant year was as high as 32.7 and 29.8% in DGF patients with and without rejections, respectively (Figure 4B).

## Discussion

The results obtained in this large multicenter cohort of more than 1,700 deceased-donor kidney transplant recipients indicate that, in addition to well-known non-immunological factors, a broad level of sensitization prior to transplantation as reflected by the co-presence of HLA class I and class II antibodies in patient's serum increases the risk of DGF development despite the currently applied sensitive antibody testing. Patients who developed DGF demonstrated impaired graft survival in the absence, and more strongly, in the presence of pre-transplant HLA antibodies or DSA. The potentiating effect of pre-transplant alloantibodies on the impact of DGF was not evident when patient survival was analyzed. In contrast, a strong influence of DGF was observed on death-censored graft loss when alloantibodies were present prior to transplantation, most probably due to additional immunological injury in an already damaged organ. This assumption was further supported by the high rate of diagnosed biopsy rejection episodes during days 8–90 after transplantation in pre-sensitized patients who had developed DGF up to day 7 post-transplant.

Mainly non-immunological donor-specific factors, such as age and brain death, and cold ischemia time have been associated with the development of DGF. In some previous studies, however, a significantly increased rate of DGF was found also in patients with pre-transplant HLA antibodies, whereas Quiroga et al. could not confirm such an association (17–20). Gibney et al. reported higher rates of primary non-function and DGF in 136 patients with pre-transplant DSA and we found in an independent previous series of 1,134 CTS Serum Study patients that no immediate function of the allograft was associated with the pre-transplant presence of especially HLA class I antibodies, whereas the association of this early adverse event with HLA class II antibodies reached statistical significance only in the univariate, but not in the multivariable analysis (18). The impact of double positivity for class I and class II on DGF development was not analyzed in this study. In two independent series of 4,136 and 5,315 kidney transplantations, the co-presence of class I and class II antibodies was found to be associated with strongly impaired graft survival (15, 21). Otten et al. reported a similar observation by analyzing the impact of pre-transplant DSA (22). The association of HLA antibodies with DGF was, however, not studied in these three studies. Peräsaari et al. analyzed 771 patients from Helsinki and found that the risk of DGF was twice as high in patients with pretransplant DSA, while pre-transplant non-DSA had no significant effect (12). In the same study the risk of DGF was increased also with broadness of sensitization, number of DSA and cumulative antibody strength. Similarly, broad pre-transplant sensitization, as indicated by the simultaneous presence of HLA class I and II antibodies, was a strong predictor that almost doubled the risk of DGF in our study, whereas, most probably due to the currently applied sensitive antibody testing, the presence of only HLA class I or only class II antibody showed no significant effect. It is assumable that the co-presence of both HLA antibody classes is reflective of a generally increased alloreactivity which, under the currently applied potent immunosuppression, can cause subclinical rejections that may go undetected in the early post-transplant phase. The rejection-mediated endothelial injury in transplant arteries could lead to a vasoconstriction, ultimately presenting the clinical picture of DGF.

In non-sensitized patients with DGF, the risk of all cause graft loss and death-censored graft loss was more than twice as high compared to the risk in patients without DGF. The risk of death-censored graft loss further increased to more than 7-fold when DGF-patients had detectable DSA pretransplant, most likely due to an increased rate of rejection episodes. Indeed, rejection was seen significantly more often and with greater severity in antibody-positive groups than in antibody-negative groups, irrespective of whether the patients developed DGF or not, while DGF alone resulted in only a small and non-significant increase in rejection episodes. Interestingly, 8 and 11% of DGF patients with or without rejection, respectively, had already lost their graft 1 year after transplantation when the donor organ was <65 years old. For recipients of an organ from a ≥65-year old donor, these figures rose to a striking 30 and 33%, respectively. This is all the more remarkable because transplant failures in the first 3 months after transplantation were not included in this calculation. Taken together, our results indicate that rejection in pre-sensitized patients is particularly harmful if they receive a pre-damaged organ from an elderly donor.

Compared to patients with no DSA and no DGF, Haller et al. found an insignificant increase of graft loss in patients with either DSA or DGF, while the same risk was 3 times and significantly higher in patients with pre-transplant DSA who developed DGF. They hypothesized that inferior graft survival in DSA-positive DGF-patients may either be due to more extensive effector functions of DSA, such as complement-activation in the inflammatory environment of DGF-patients compared to patients without DGF, or overlooked rejection episodes during the DGF process causing increased harm to the allograft (13). According to our data, a complementary explanation for the observed inferior outcomes in DSA-positive patients with DGF might be the occurrence of rejections in an allograft that already has been damaged by DGF. DGF-associated damage can predispose the graft to an increased risk of immune attack by upregulating major histocompatibility complex as well as non-major histocompatibility complex alloantigens in the graft. Furthermore, graft injury caused by brain death or early damage due to DGF can lead to the production of chemokines that attract immune cells into the graft and eventually result in rejections. Given the high 36% rate of DGF in patients who received an organ from a ≥65-year-old donor in our study and the inferior outcomes, careful selection of recipients of these organs during organ allocation is mandatory, especially when they are presensitized.

The strength of the study is, besides the high patient number, the existence of relevant non-immunological and immunological variables, in all patients as, due to study design, only patients on whom these variables were available were analyzed. Limitations of the study are the multicenter approach, which forced us to reduce the number of variables that could be asked to the most relevant ones, and the missing information on the presence of pre-transplant DSA in 56% of the patients. Moreover, in these patients the DSA information was delivered by the participating centers and there is heterogeneity not only in the determination but also in consideration of acceptable levels of DSA. Single antigen tests used for DSA testing stem from two different suppliers with slight differences in the sensitivity and composition of detected HLA antibody specificities and there are technical variations between the laboratories, e.g., in pretreatment of sera to eliminate the prozone effect. In addition, the centers are using different algorithms for the determination of unacceptable HLA antigen mismatches, and depending on the algorithm, more or fewer organ offers are excluded for patients with a similar antibody profile (23, 24). Overall, despite these limitations, this is the first large-scale study that demonstrates the alloantibody-dependent detrimental influence of DGF on post-transplant outcomes in the modern era of transplantation.

**Figure 5:**
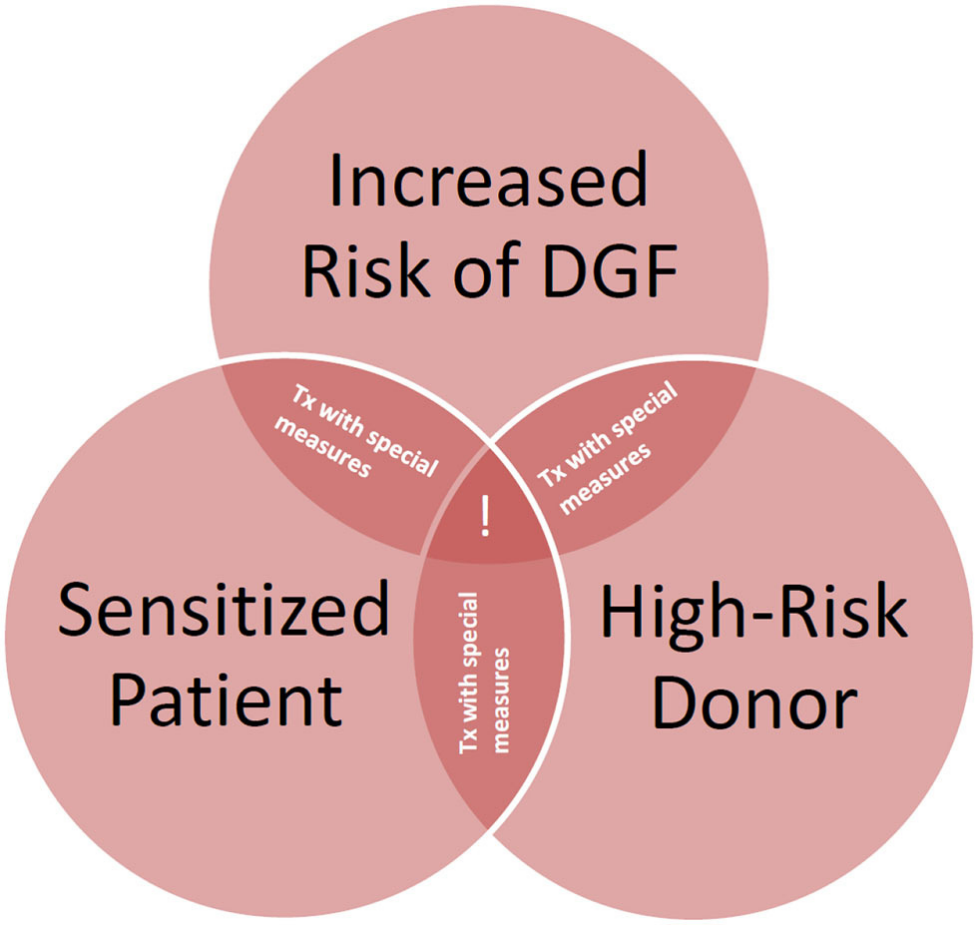
Need for measures to improve graft survival in high-risk recipients of deceased donor kidney transplants. Special measures = avoidance of prolonged cold ischemia, avoidance of non-acceptable human leukocyte antigen (HLA) mismatches also in elderly recipients, desensitization if pre-sensitized, post-transplant HLA antibody monitoring. !, consideration of alternative options; Tx, transplantation.

In conclusion, DGF has a strong influence on graft survival, also in the absence of pre-transplant HLA alloantibodies. However, pre-transplant HLA alloantibodies are a predisposing factor for DGF, and the presence of alloantibodies, especially that of DSA, together with DGF are associated with strongly impaired graft outcome. Adequate measures to prevent DGF in sensitized patients should be in place, especially during the allocation and transplantation of organs from elderly donors (Figure 5).

## Data Availability Statement

The raw data are available upon request to the Collaborative Transplant Study in accordance with the consents of the patients, the participating transplant centers and registries.

## Ethics Statement

The studies involving human participants were reviewed and approved by Ethics committee of Heidelberg University. The patients/participants provided their written informed consent to participate in this study.

## Author Contributions

CS, BD, and CM designed the study, analyzed the data and wrote the paper. BD performed the statistical analysis. AR contributed to data acquisition. FK, LS, FEc, VS, SŽ-Ć, NK, DK, PB, MH, MZi, MN, FEm, PP, HK, RW, AM, and MZe delivered the sera and clinical data and contributed to the writing of the paper. CS, TT, and SS participated in testing of sera from the Heidelberg transplant center. The study received no external funding.

## Conflict of Interest

The authors declare that the research was conducted in the absence of any commercial or financial relationships that could be construed as a potential conflict of interest.
